# Single-cell transcriptomics and machine learning characterize LTA4H-linked lipid metabolism and inflammatory signatures in the knee osteoarthritis IPFP/synovium compartment

**DOI:** 10.1371/journal.pone.0360615

**Published:** 2026-10-12

**Authors:** Xin Chen, Shudong Li, Xiaole Wang, Wanyang Chen, Shangfeng Qi, Jifeng Zhao

**Affiliations:** 1 Affiliated Hospital of Shandong University of Traditional Chinese Medicine, Jinan, Shandong, China; 2 The First Clinical Medical College, Shandong University of Traditional Chinese Medicine, Jinan, Shandong, China; 3 The Fifth Clinical Medical College, Guangzhou University of Chinese Medicine, Guangzhou, Guangdong, China; University of Vermont College of Medicine, UNITED STATES OF AMERICA

## Abstract

Knee osteoarthritis (KOA) is a whole-joint disease characterized by inflammation, immune dysregulation, and metabolic imbalance. To define lipid metabolism-related transcriptional heterogeneity within the infrapatellar fat pad (IPFP)/synovium compartment, we integrated publicly available single-cell transcriptomic data from IPFP/synovium-derived joint cells with bulk synovial transcriptomic datasets from patients with KOA and controls. Lipid metabolism-related scores were derived using multiple single-cell scoring algorithms, and candidate genes were identified through score-associated analyses, differential expression, protein–protein interaction analysis, and five machine-learning approaches, followed by external bulk validation and single-cell characterization. Myeloid cells showed the most consistent KOA-associated increases in lipid metabolism-related transcriptional scores. Fifty composite-score-associated genes were identified and were enriched in lipid metabolic, lysosomal, and PPAR-related pathways. Cross-screening prioritized PLIN2, LTA4H, and PPT1, with LTA4H showing the strongest association with lipid metabolism-related transcriptional scores. LTA4H was predominantly enriched in myeloid cells, particularly monocytes and inflammatory macrophages, and was increased in KOA myeloid cells and high-score cells. Cell–cell communication, myeloid subclustering, and pseudotime analyses linked LTA4H-positive myeloid cells to inflammatory signaling and the transition from monocytes toward inflammatory macrophages. In silico knockout analysis further implicated LTA4H in regulatory programs related to leukocyte migration, chemotaxis, phagocytosis, and cytokine receptor binding. In a rat KOA model, LTA4H protein expression was increased in knee synovial tissues and spatially colocalized with CD68-positive macrophages. These findings define a myeloid-cell-centered context for LTA4H in KOA and link its expression to lipid metabolism-related transcriptional features and inflammatory myeloid-cell states.

## Introduction

Knee osteoarthritis (KOA) is a common chronic degenerative joint disease whose onset and progression are driven by multiple factors, including abnormal mechanical loading, ageing and metabolic alterations [1–3]. Its pathological process involves not only articular cartilage degeneration but also synovitis, subchondral bone remodeling and abnormalities of periarticular soft tissues, reflecting an inflammation–structure coupling pattern characteristic of a whole-joint disease [4]. Recent evidence suggests that KOA is not merely a disorder of mechanical wear, but a complex pathological condition accompanied by substantial metabolic dysregulation. Small-molecule metabolites can promote inflammatory responses and tissue remodeling by participating in cellular signal transduction, immune regulation and interactions within the local microenvironment [5,6]. Among these processes, aberrant lipid metabolism plays an important role in the development and progression of KOA. Imbalances in fatty acid metabolism, abnormal synthesis of lipid mediators and lipid oxidative stress may affect immune-cell activation, inflammatory cytokine release and extracellular matrix degradation, thereby aggravating joint inflammation, synovial hyperplasia and pain progression [7–9]. However, the cellular subsets that drive lipid metabolic abnormalities in KOA synovial tissue, as well as the key regulatory genes and cell type-specific mechanisms involved, remain to be systematically elucidated.

The synovium is an essential component of the knee joint and consists of synovial fibroblasts, macrophages, endothelial cells and other stromal and immune cell populations. It plays a critical role in maintaining joint homeostasis, secreting synovial fluid and regulating the local immune microenvironment [10,11]. In KOA, the synovium commonly exhibits varying degrees of inflammatory cell infiltration, angiogenesis, fibrosis and synovial hyperplasia, providing an important pathological basis for local inflammatory amplification and cartilage degeneration [12–14]. Previous studies have suggested that the synovium is not only a major site of inflammatory responses but also an active participant in lipid metabolic dysregulation [15]. Aberrant lipid metabolism may influence the inflammatory phenotype, secretory function and intercellular communication of synovial cells, thereby reshaping the joint microenvironment and promoting disease progression [16,17]. However, because synovial tissue comprises multiple cell types and displays marked cellular heterogeneity, conventional bulk transcriptomic analysis cannot accurately resolve cell subset-specific alterations in lipid metabolic programs or their regulatory networks, thereby limiting the identification of cell-type-specific molecular features.

Single-cell RNA sequencing (scRNA-seq) enables the characterization of tissue cellular composition, state transitions and cell-subset heterogeneity at single-cell resolution, and allows the identification of cell type-specific differential expression and functional programs [18]. It therefore provides an important tool for investigating lipid metabolic reprogramming in KOA synovium. However, single-cell data are inherently high-dimensional and noisy, and differential expression analysis alone is often insufficient to identify robust candidate genes with strong generalizability. Machine-learning approaches enable feature selection and model construction in complex multi-feature settings, facilitating the identification of disease-relevant gene combinations from transcriptomic data and improving the robustness and interpretability of candidate gene prioritization [19,20]. Therefore, based on single-cell transcriptomic data from IPFP/synovium-derived joint cells from patients with KOA and healthy controls in the GEO database, this study systematically characterized lipid metabolism-related transcriptional features and their cell-type heterogeneity within the KOA-associated IPFP/synovium compartment. We first integrated lipid metabolism-related gene-set scoring, correlation analysis, differential expression analysis and PPI network analysis to identify candidate genes associated with the composite lipid metabolism-related transcriptional score. We then incorporated independent bulk transcriptomic datasets and multiple machine-learning methods to screen KOA-related feature genes, followed by single-cell expression mapping and functional analyses to prioritize candidate genes with potential disease relevance. Finally, we explored their cell-type-specific characteristics and potential biological relevance in terms of cellular origin, expression patterns, cell–cell communication, pseudotime dynamics and in silico knockout perturbation. This study provides an integrative transcriptomic and experimental basis for understanding the cellular context and transcriptional features associated with lipid metabolic abnormalities within the KOA-associated IPFP/synovium compartment.

## Materials and methods

### Data acquisition

Single-cell RNA-sequencing data were obtained from the Gene Expression Omnibus (GEO; https://www.ncbi.nlm.nih.gov/geo/) under accession number GSE216651 [21]. According to the original GEO metadata, these samples were annotated as joint-derived “IPFP/synovium cells.” In addition, multiple bulk transcriptomic datasets of synovial tissues, including GSE55235, GSE55457 [22] and GSE82107 [23], were downloaded from GEO and used as the training cohort for further candidate-gene screening. Two additional bulk transcriptomic datasets, GSE1919 [24] and GSE206848, were combined to construct the external validation cohort. The training cohort comprised 30 KOA samples and 27 control synovial samples, whereas the validation cohort included 12 KOA samples and 12 control synovial samples. Detailed sample-level information, including GEO/GSM accession numbers, group annotations, tissue sources, inclusion or exclusion status, exclusion reasons, and analytical purposes, is provided in S1 Table. Lipid metabolism-related gene sets were obtained from the Molecular Signatures Database (MSigDB; https://www.gsea-msigdb.org), from which 756 lipid metabolism-related genes (LMRGs) were curated after preprocessing (S2 Table).

### scRNA-seq data analysis

The scRNA-seq data were processed using the Seurat package. Individual 10X Genomics expression matrices were merged, and low-quality cells were removed based on mitochondrial gene percentage <30%, hemoglobin gene percentage <5%, detected genes (200–5,000), and UMI counts (200–30,000). Doublets were identified using DoubletFinder with an expected doublet rate of 7.5% and excluded. The filtered data were normalized using LogNormalize with a scale factor of 10,000, followed by identification of 2,000 highly variable genes using the vst method. Principal component analysis (PCA) was performed after scaling, and Harmony was applied using sample origin as the batch variable to correct sample-related batch effects. Clustering and UMAP visualization were performed using the first 20 Harmony-corrected dimensions, with the resolution parameter in FindClusters set to 0.1. Marker genes were identified from normalized RNA expression using FindAllMarkers with the Wilcoxon rank-sum test and a log2FC threshold of 0.25; genes with adjusted P < 0.05 were retained for cell-type annotation based on canonical marker genes (S3 Table). Harmony-corrected dimensions were used for clustering and visualization rather than differential expression analysis.

To characterize lipid metabolism-related transcriptional features, five algorithms, including AUCell, UCell, singscore, ssGSEA, and AddModuleScore, were used to calculate lipid metabolism-related transcriptional scores at single-cell resolution. All five methods were applied to the same scRNA-seq expression dataset using the same REACTOME lipid metabolism gene set restricted to genes detected in the RNA expression data. Each algorithm-specific score was Z-standardized across all cells, and the five standardized scores were combined using an unweighted arithmetic mean to generate a composite lipid metabolism-related transcriptional score. As sensitivity analyses, pairwise Spearman correlations were assessed among the five scoring methods and between the individual or composite scores and technical variables (nCount_RNA, nFeature_RNA, and mitochondrial transcript percentage); disease- and cell-type-associated patterns were also examined separately for each scoring method at the sample level. Cells were then divided into high- and low-score groups according to the overall median composite score across all cells. In KOA cells, Spearman correlation analysis was used to identify the top 100 lipid metabolism-related genes most positively associated with the composite score. Differential expression analysis between the high- and low-score groups was performed on RNA expression using FindMarkers with default settings, and genes with adjusted P < 0.05 and avg_log2FC > 0.5 were considered upregulated. The intersection of the top 100 correlated genes and upregulated differentially expressed genes defined the composite-score-associated candidate genes, which were subjected to Gene Ontology (GO) and Kyoto Encyclopedia of Genes and Genomes (KEGG) enrichment analyses. Finally, a protein–protein interaction (PPI) network was constructed using STRING v12.0 for Homo sapiens, with a minimum interaction confidence score of 0.400. Genes without identified interactions were excluded from degree calculation. The top 20 genes ranked by node degree were retained for subsequent machine-learning analysis, while the top 30 genes were displayed for visualization. Betweenness, closeness, and eigenvector centrality were additionally evaluated as sensitivity analyses of degree-based gene prioritization.

### Bulk transcriptomic expression data processing

Bulk transcriptomic expression matrices and sample grouping information were downloaded using the GEOquery package. GSE55235, GSE55457, and GSE82107 were merged as the training cohort, whereas GSE206848 and GSE1919 were merged as the external validation cohort. For microarray-based datasets, probe IDs were converted into gene symbols according to the corresponding platform annotation files. When multiple probes mapped to the same gene, their average expression value was used to represent the expression level of that gene. Common genes retained across the merged datasets were then used to construct the training and validation expression matrices. The expression matrices were normalized using the limma package, and batch effects among different datasets were corrected using the ComBat function in the sva package, while preserving the KOA and control group information in the model matrix. The final expression matrices matched with clinical grouping information were used for subsequent machine-learning-based feature selection, model construction, and external validation.

### Machine-learning-based feature selection and model construction

Based on the core candidate genes identified from the PPI network, five machine-learning-based feature-selection methods, including LASSO, elastic net, random forest, Boruta, and SVM-RFE, were applied to the training cohort to identify KOA-related candidate genes. LASSO and elastic net logistic regression were implemented using glmnet with α = 1 and 0.5, respectively, and λ was selected by 10-fold cross-validation using lambda.min. Random forest was performed with 2,000 trees, retaining the top 15 genes ranked by MeanDecreaseGini. Boruta was run with maxRuns = 500, and Confirmed features were retained. SVM-RFE was implemented using a linear-kernel SVM (C = 1) with 5-fold cross-validation, and the feature subset with the best cross-validated performance was selected. No class balancing or repeated/nested cross-validation was applied. Full parameter settings, random seeds, and feature-selection criteria are provided in S4 Table. Genes selected by all five methods were defined as consensus candidate feature genes. PLIN2, LTA4H, and PPT1 were subsequently entered into a multivariable logistic regression model fitted in the training cohort, and the complete model formula and coefficients are provided in S4 Table. Receiver operating characteristic (ROC) analysis was used to evaluate the discriminatory performance of the three-gene model in the training and external validation cohorts. Expression differences in the candidate genes between KOA and control samples were further examined. Reactome GSEA was performed separately for PLIN2, LTA4H, and PPT1 in the bulk transcriptomic training cohort by stratifying samples into high- and low-expression groups according to the median expression of each gene and ranking genes by limma-derived log2 fold-change values. Preranked GSEA was conducted using clusterProfiler with the MSigDB C2:CP:REACTOME collection (version 2026.1.Hs), with gene-set sizes of 10–500 genes; significance was estimated using the fgsea implementation, and pathways with Benjamini–Hochberg-adjusted P < 0.05 were considered significant.

### Single-cell expression characterization of candidate feature genes and prioritization of the key candidate gene

To prioritize key targets from the candidate feature genes identified by machine learning, their single-cell expression profiles were analyzed using the annotated scRNA-seq dataset. UMAP feature plots, violin plots, and dot plots were first used to visualize the expression distribution of candidate genes across major cell types and disease groups. Cells were then divided into high- and low-score groups according to the median composite lipid metabolism score, and candidate gene expression was compared between the two groups to assess its association with the lipid metabolism-related transcriptional score. Spearman correlation analysis was further performed in KOA myeloid cells to evaluate the relationship between candidate gene expression and the composite lipid metabolism score. Based on these analyses, LTA4H was ultimately prioritized as the key candidate gene for subsequent investigation.

### Cell–cell communication analysis of LTA4H-associated myeloid cells

After LTA4H was prioritized for further cell-type-specific characterization, subsequent cell–cell communication analysis focused on myeloid cells, the major cell population expressing LTA4H. In KOA samples, myeloid cells were divided into LTA4H⁺Myeloid and LTA4H^−^Myeloid groups according to LTA4H expression status; myeloid cells with LTA4H expression greater than 0 were defined as LTA4H⁺Myeloid. Ligand–receptor interactions among LTA4H⁺Myeloid cells, LTA4H^−^Myeloid cells, and other cell types were then analyzed using the CellChat package. The Secreted Signaling category from the CellChatDB human database was used, and cell populations containing fewer than 10 cells were excluded. To reduce computational burden and limit differences in group size, cell populations were randomly downsampled to a maximum of 2,000 cells per group before CellChat analysis. Communication probabilities were calculated, significant signaling pathways were identified, and the cell–cell communication network was aggregated. Finally, communication network plots, signaling input/output pattern plots, and bubble plots were used to visualize the communication characteristics of LTA4H-associated myeloid cells in the KOA synovial microenvironment.

### Pseudotime trajectory analysis of myeloid cells

Pseudotime trajectory analysis of myeloid cells was performed using Monocle 2. Myeloid cells were first extracted from the Seurat object, and a CellDataSet object was constructed using the UMI count matrix from the RNA assay. Size factors and dispersion were then estimated, and ordering genes were selected based on differentially expressed genes among myeloid-cell subclusters. Dimensionality reduction was performed using the DDRTree method, and the State with a higher proportion of control cells was set as the starting state to construct the pseudotime trajectory. Disease group, LTA4H expression status, and LTA4H expression levels were further mapped onto the trajectory to examine the distribution patterns of LTA4H⁺Myeloid and LTA4H^−^Myeloid cells during pseudotime progression. In addition, the proportion of LTA4H-positive cells in each State was calculated to assess the association between LTA4H expression and myeloid-cell state transitions.

### Myeloid-cell subclustering and inflammatory trajectory analysis

To characterize myeloid-cell heterogeneity, myeloid cells were extracted from the annotated scRNA-seq dataset and reprocessed using Seurat, including normalization, PCA-based dimensionality reduction, UMAP visualization, and unsupervised clustering. Marker genes for each subcluster were identified from the RNA assay using FindAllMarkers with the Wilcoxon rank-sum test, min.pct = 0.25, and logfc.threshold = 0.25; genes with adjusted P < 0.05 were retained for annotation. Differences in myeloid-cell subcluster composition between the control and KOA groups were then compared, and the expression distribution of LTA4H across subclusters was analyzed. Monocytes and inflammatory macrophages were further selected for pseudotime analysis using Monocle 2. A CellDataSet object was constructed based on the UMI count matrix from the RNA assay, and ordering genes were selected according to differentially expressed genes between the two cell populations. Dimensionality reduction was performed using the DDRTree method, and the State with the highest proportion of monocytes was set as the trajectory starting point. Cell subcluster identity, disease group, LTA4H expression level, and LTA4H expression status were then mapped onto the trajectory. An inflammatory score was calculated based on CCL3, TNF, IL1B, and OLR1 to evaluate changes in inflammatory status along pseudotime, and the proportion of LTA4H-positive cells at different pseudotime stages was quantified.

### In silico knockout analysis of LTA4H

To evaluate the potential influence of LTA4H on the regulatory network of inflammatory macrophages, single-gene in silico knockout analysis was performed using scTenifoldKnk. Inflammatory macrophages were first extracted from myeloid cells, and the UMI count matrix from the RNA assay was used as input. To ensure analytical stability, genes expressed in at least 10 cells were retained, and LTA4H together with inflammation-related marker genes were included in the analysis. scTenifoldKnk was then used to construct gene regulatory networks under the baseline and simulated LTA4H-knockout states, followed by manifold alignment to assess network perturbations induced by LTA4H knockout. The degree of perturbation for each gene was represented by the differential regulation Z-score, with positive and negative values indicating the direction of differential regulation. Perturbed genes after simulated LTA4H knockout were further selected and subjected to Gene Ontology (GO) enrichment analysis using the clusterProfiler package, including biological process, cellular component, and molecular function categories. Adjusted p < 0.05 was considered the threshold for significant enrichment.

### Animal experiments

Eight-week-old male Sprague–Dawley (SD) rats weighing 220–260 g were obtained from the Animal Experimental Center of Shandong University of Traditional Chinese Medicine. The rats were randomly assigned to a sham-operated group (Sham, n = 4) or a knee osteoarthritis group (KOA, n = 4). Anesthesia was induced and maintained with 2.0–2.5% inhaled isoflurane at an oxygen flow rate of 0.5–0.7 L/min. The KOA model was established using a modified Hulth procedure. After anesthesia, the left knee was shaved and disinfected, and the joint structures were exposed through a medial parapatellar approach. The medial collateral ligament and anterior cruciate ligament were transected, and the medial meniscus was removed. The joint cavity was subsequently irrigated with sterile saline, and the incision was closed layer by layer. Rats in the Sham group underwent the same surgical exposure without ligament transection or meniscectomy, followed by irrigation and wound closure. All rats were allowed unrestricted activity after surgery. Six weeks after model induction, the rats were euthanized by intraperitoneal injection of sodium pentobarbital (200 mg/kg). After death was confirmed, knee joint tissues were promptly collected for subsequent experiments. All animal procedures were approved by the Ethics Committee of the Affiliated Hospital of Shandong University of Traditional Chinese Medicine (approval no. SDSZYYAWE20240801001).

### Histological analysis

Knee joint tissues were fixed in 4% paraformaldehyde for 72 h and decalcified in 10% EDTA for 4 weeks. The tissues were then routinely dehydrated, cleared, paraffin-embedded, and sectioned at a thickness of 5 μm. Safranin O/Fast Green staining was performed to evaluate histopathological changes in articular cartilage. Cartilage degeneration was assessed using the OARSI histopathological scoring system for rat osteoarthritis. The medial tibial plateau in each section was divided into three regions, each of which was scored separately, and the three regional scores were summed to obtain the total score for that section. Three standardized sections were evaluated for each animal, and the mean of the three section scores was used as the final OARSI score for statistical analysis. Individual animals were considered independent biological replicates.

### Immunohistochemistry

Paraffin-embedded tissue sections were deparaffinized, rehydrated, subjected to antigen retrieval, and blocked before incubation with a primary antibody against LTA4H (Bioss, bs-3908R, 1:300) at 4°C overnight. The following day, the sections were incubated with an HRP-conjugated secondary antibody for 1 h at room temperature, followed by visualization with 3,3′-diaminobenzidine (DAB) and counterstaining with hematoxylin. After dehydration, clearing, and mounting, the sections were examined and imaged under a microscope. LTA4H immunohistochemical staining was semiquantitatively assessed using Saiviewer software by calculating the H-score. Three standardized synovial regions were analyzed for each animal, and the mean H-score of the three regions was used as the final animal-level value for statistical analysis.

### Tissue immunofluorescence

Paraffin-embedded tissue sections were incubated in a 39°C oven, deparaffinized in xylene, and rehydrated through a graded ethanol series. Antigen retrieval was subsequently performed using a pepsin-based retrieval solution. After blocking with 3% bovine serum albumin (BSA), the sections were incubated overnight at 4°C with mouse anti-CD68 (HA601115, 1:300) and rabbit anti-LTA4H (Bioss, bs-3908R, 1:300) primary antibodies. The following day, appropriate fluorescence-conjugated secondary antibodies corresponding to the host species of the primary antibodies were applied and incubated at room temperature in the dark. Nuclei were counterstained with DAPI, and fluorescence images were acquired using a fluorescence microscope after mounting. Spatial colocalization of LTA4H and CD68 signals in knee synovial tissues was evaluated based on their fluorescence distribution.

### Statistical analysis

Statistical analyses were performed using SPSS version 25.0. Individual animals were considered independent biological replicates (n = 4 per group), and measurements obtained from multiple sections or regions of the same animal were averaged before between-group comparisons. OARSI scores were compared using the Mann–Whitney U test, whereas LTA4H immunohistochemical H-scores were compared using an independent-samples t-test. P < 0.05 was considered statistically significant. Graphs were generated using GraphPad Prism version 8.4.3. The raw animal-level data underlying the OARSI and LTA4H H-score analyses are provided in S5 Table.

## Results

### scRNA-seq analysis of the IPFP/synovium compartment in KOA

The overall workflow of this study is shown in Fig 1. We first performed quality control, doublet removal, and normalization of the scRNA-seq data, resulting in 63,642 high-quality cells for downstream analysis. Quality-control metrics showed that the number of detected genes per cell, UMI counts, mitochondrial gene percentage, and hemoglobin gene percentage were generally stable across samples, indicating that the data quality was suitable for subsequent analyses (Fig 2A). Harmony was then applied to correct batch effects across samples. UMAP visualization showed that cells from different samples were well mixed in the corrected low-dimensional space, without obvious separation driven by sample origin (Fig 2B). Unsupervised clustering based on the Harmony-corrected data identified seven cell clusters (Fig 2C). By integrating the expression patterns of canonical marker genes, these cells were annotated into six major cell types, including fibroblasts, endothelial cells, mural cells, myeloid cells, lymphocytes, and mast cells (Fig 2D). Dot plot analysis showed that representative marker genes of each cell type were specifically enriched in the corresponding cell populations, supporting the reliability of cell-type annotation (Fig 2E and 2F). Further comparison of cellular composition across samples and disease groups showed that all major cell types were represented in both the control and KOA groups, although their proportions varied across samples and groups (Fig 2G and 2H). Group-stratified UMAP visualization further confirmed that both the control and KOA groups covered the major cell populations (Fig 2I). In addition, functional annotation analysis based on marker genes of each cell type revealed enrichment patterns consistent with their biological identities. For example, endothelial cells were mainly enriched in angiogenesis- and endothelial cell differentiation-related processes, fibroblasts were enriched in extracellular matrix organization and cell adhesion-related processes, and myeloid cells were enriched in leukocyte activation and immune response-related processes, further supporting the validity of the cell-type annotations (Fig 2J).

**Fig 1:**
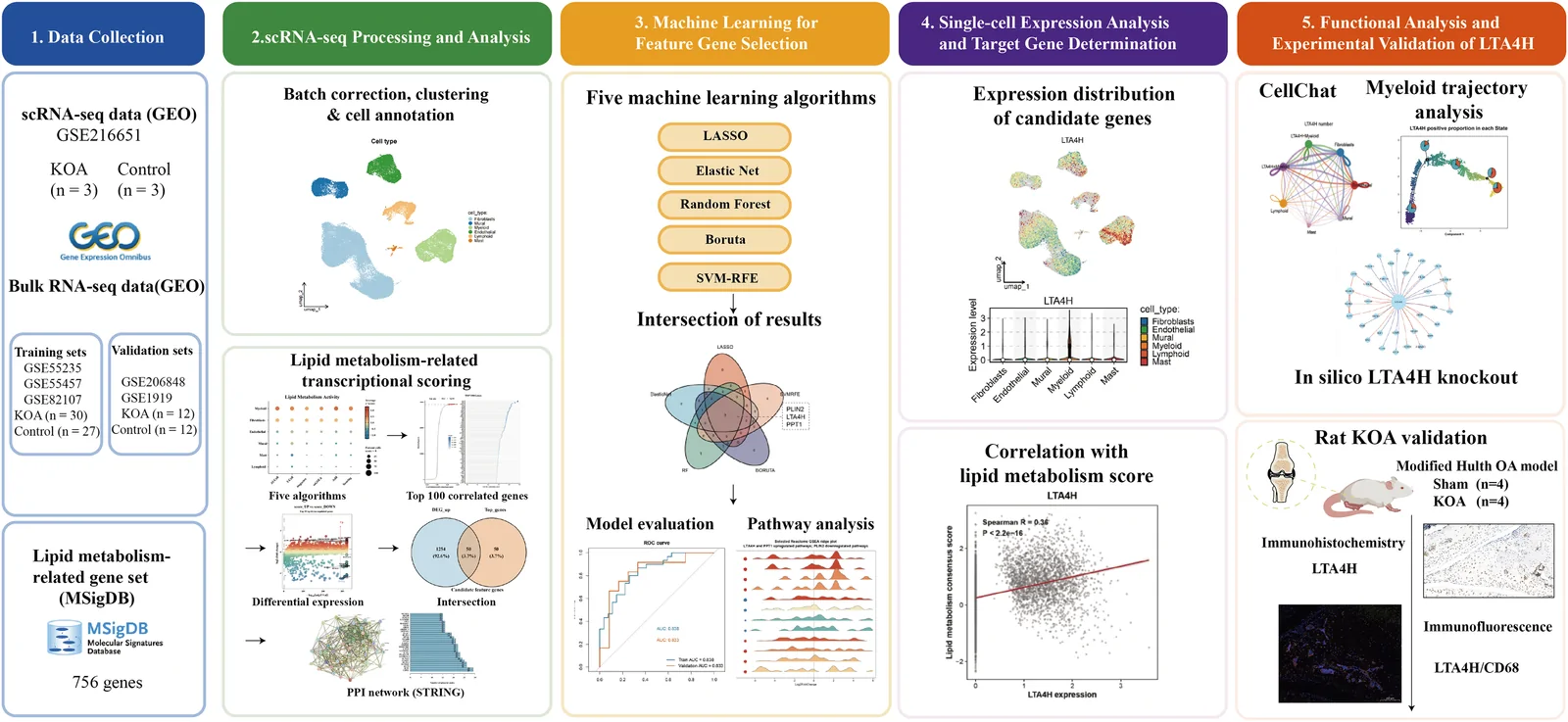
Workflow diagram of the study.

**Fig 2:**
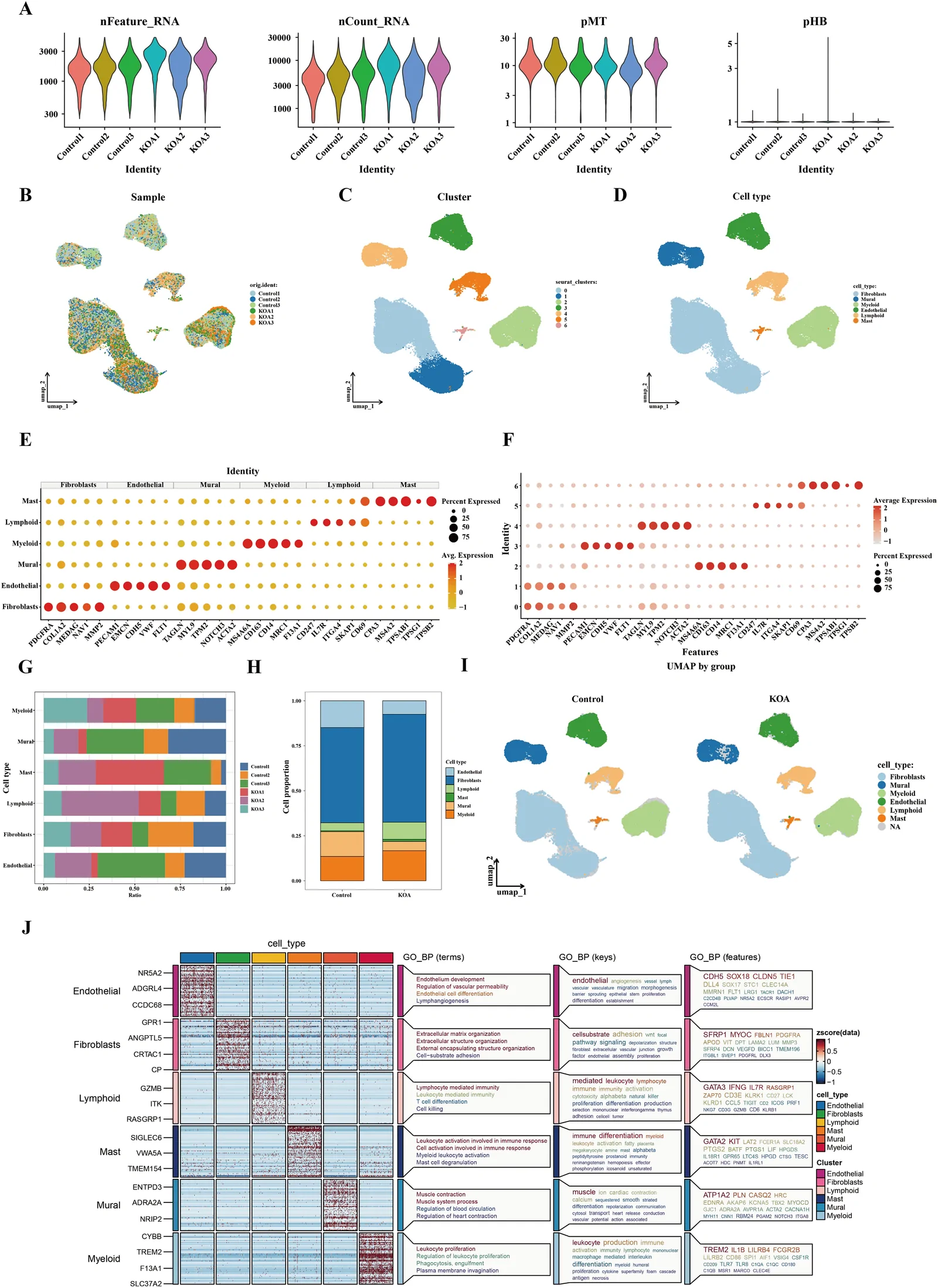
Quality control, clustering, annotation, and compositional analysis of IPFP/synovium-derived cell subpopulations. (A) Quality-control results of the scRNA-seq dataset, including the number of detected genes per cell (nFeature_RNA), UMI counts (nCount_RNA), mitochondrial gene percentage (pMT), and hemoglobin gene percentage (pHB); (B) UMAP distribution after Harmony-based correction of batch effects across different samples; (C) UMAP plot of cell clusters generated by unsupervised clustering based on Harmony-corrected data; (D) Cell type annotation identifying six major cell types; (E) Dot plot showing the expression of canonical marker genes across different cell types; (F) Dot plot showing the expression of canonical marker genes across different cell clusters; (G) Relative compositional proportions of cell types across different samples; (H) Relative compositional proportions of cell types between the control and KOA groups; (I) UMAP distribution of major cell types in the control and KOA groups; (J) Heatmap of marker gene expression across cell types and corresponding Gene Ontology biological process enrichment results. Six biological samples (three KOA and three controls; 63,642 cells after quality control) were analyzed.

### Lipid metabolism-related transcriptional scoring in the scRNA-seq data

To characterize lipid metabolism-related transcriptional features at single-cell resolution, we scored the REACTOME lipid metabolism gene set using five algorithms, including AUCell, UCell, singscore, ssGSEA, and AddModuleScore, and integrated these results into a composite lipid metabolism score. The lipid metabolism-related scores differed substantially across cell types, with relatively higher scores observed in myeloid cells and fibroblasts and lower scores in lymphocytes (Fig 3A and 3B). UMAP visualization further showed that cells with high lipid metabolism scores were mainly distributed in regions corresponding to myeloid cells and fibroblasts (Fig 3C). At the single-cell level, the overall composite lipid metabolism score was higher in the KOA group than in the control group (Wilcoxon test, p < 2.2 × 10^ − 16), and cells with high scores were more widely distributed in the KOA group (Fig 3D and 3E). Stratified analysis by cell type showed that lipid metabolism scores tended to increase in fibroblasts, mast cells, and myeloid cells in the KOA group, whereas lymphocytes showed decreased scores, suggesting cell type-specific lipid metabolism-related transcriptional alterations within the analyzed IPFP/synovium compartment (Fig 3F).

**Fig 3:**
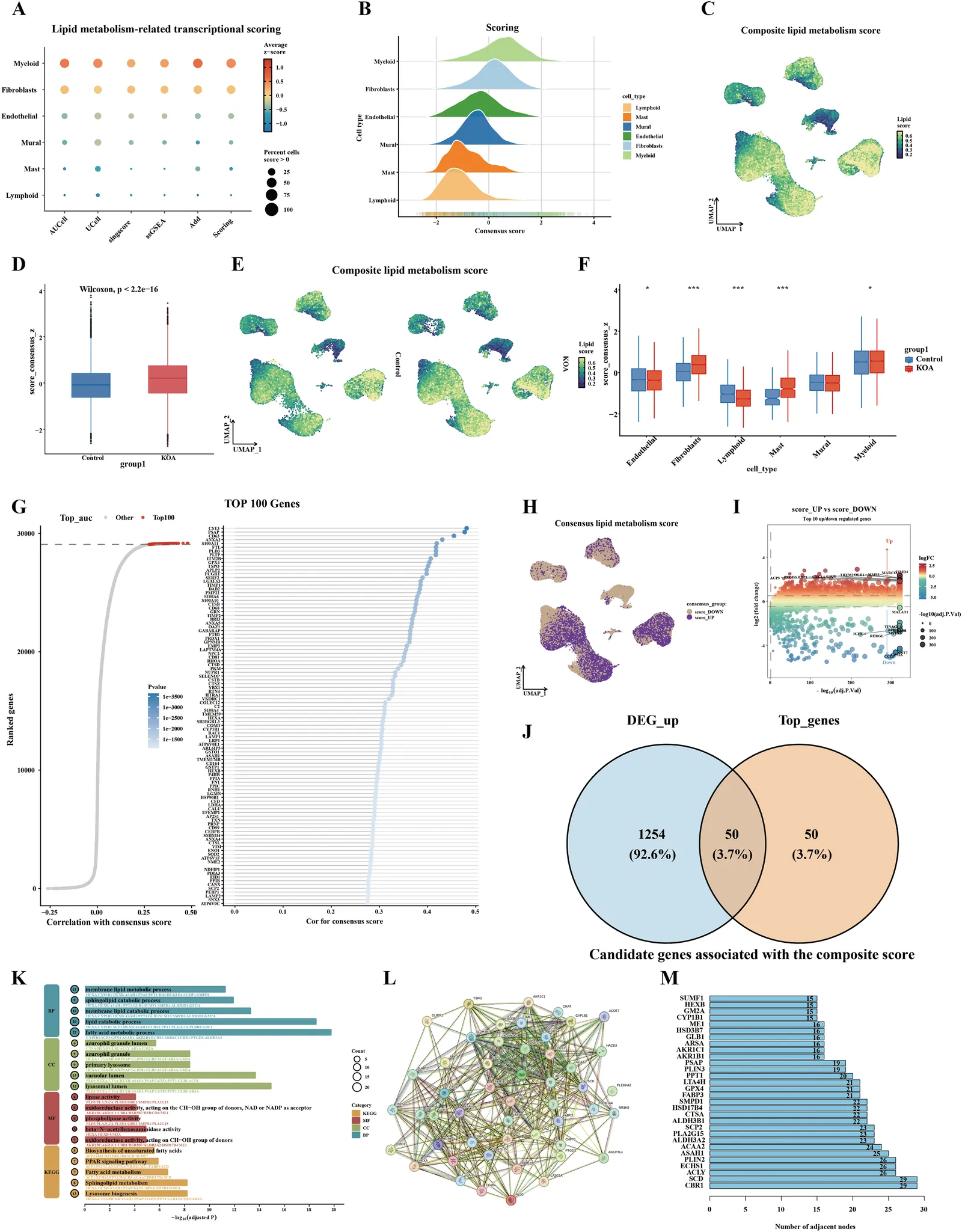
Lipid metabolism-related transcriptional scoring in KOA and screening and functional enrichment analysis of related candidate genes. (A) Distribution of lipid metabolism-related gene-set scores across different cell types evaluated using five scoring algorithms; (B) Distribution of the composite lipid metabolism score across different cell types; (C) UMAP distribution of the composite lipid metabolism score; (D) Comparison of the overall composite lipid metabolism score between the control and KOA groups; (E) UMAP distribution of the composite lipid metabolism score in the control and KOA groups; (F) Comparison of the composite lipid metabolism score across cell types between the control and KOA groups; (G) Spearman correlation analysis between lipid metabolism-related gene expression and the composite score in KOA samples, with screening of the top 100 correlated genes; (H) UMAP distribution of score_UP and score_DOWN cells classified according to the median composite lipid metabolism score; (I) Differential expression analysis between the score_UP and score_DOWN groups; (J) Intersection analysis of upregulated differentially expressed genes in the score_UP group and the top 100 correlated genes; (K) GO and KEGG functional enrichment analysis of the intersecting genes; (L) Protein–protein interaction network analysis of the intersecting genes; (M) Node-degree analysis of core genes in the PPI network. The scRNA-seq dataset comprised six biological samples and 63,642 cells; correlations are reported as Spearman’s ρ.

The five algorithm-specific scores were positively but heterogeneously correlated, with pairwise Spearman correlation coefficients ranging from 0.166 to 0.982 (S1A Fig). The composite score was positively correlated with nCount_RNA (ρ = 0.725) and nFeature_RNA (ρ = 0.753), whereas its correlation with mitochondrial transcript percentage was weaker and negative (ρ = −0.182) (S1B Fig). At the sample level, four of the five individual methods showed higher median scores in KOA than in controls, whereas UCell showed the opposite overall direction (S1C Fig). Cell-type-stratified analysis showed concordant KOA-associated increases across all five methods in myeloid and mast cells and concordant decreases in lymphoid cells; fibroblasts showed higher scores with four of five methods, whereas endothelial and mural cells displayed less consistent method-dependent patterns (S1D Fig). Thus, the principal cell-type-associated transcriptional patterns showed partial concordance across scoring methods, with algorithm-specific variation.

Spearman correlation analysis was further performed in KOA cells to identify the top 100 genes most positively correlated with the composite lipid metabolism score (Fig 3G). Cells were then divided into score_UP and score_DOWN groups according to the median composite score. The score_UP cells were mainly localized to cell population regions with relatively high lipid metabolism-related transcriptional scores (Fig 3H). Differential expression analysis identified a large number of upregulated genes in the score_UP group (Fig 3I). By intersecting these upregulated genes with the top 100 correlated genes, 50 composite-score-associated candidate genes were identified (Fig 3J). Functional enrichment analysis showed that these genes were mainly enriched in Gene Ontology terms related to membrane lipid metabolism, sphingolipid metabolism, lipid catabolism, fatty acid metabolism, lysosome-associated structures, and lipase and phospholipase activities. KEGG pathway analysis further revealed significant enrichment in the PPAR signaling pathway, fatty acid metabolism, fatty acid degradation, sphingolipid metabolism, and lysosome pathways (Fig 3K). PPI network construction and node-degree analysis showed that genes such as CBR1, SCD, ACLY, ECHS1, PLIN2, ASAH1, ACAA2, and ALDH3A2 had relatively high connectivity within the network (Fig 3L and 3M). The top 20 genes ranked by node degree were retained for subsequent machine-learning analysis, while the top 30 genes were displayed in the degree-ranking plot. Together, these findings indicate cell-type-dependent differences in lipid metabolism-related transcriptional scores in KOA, with the most consistent KOA-associated increases observed in myeloid cells and broadly higher scores in fibroblasts across most scoring methods. Alternative centrality measures showed strong concordance with the degree-based ranking, with top-20 overlaps of 18/20, 20/20, and 19/20 for betweenness, closeness, and eigenvector centrality, respectively. The 50 composite-score-associated candidate genes identified here may participate in lipid metabolism-related transcriptional remodeling within the KOA-associated IPFP/synovium compartment.

### Machine-learning-based screening of KOA-related candidate feature genes

Based on the top 20 composite-score-associated core candidate genes ranked by node degree in the PPI network, five machine-learning algorithms, including LASSO, elastic net, random forest, Boruta, and SVM-RFE, were further applied to the bulk transcriptomic training cohort to identify KOA-related candidate feature genes (Fig 4A–4E). By intersecting the genes selected by all five algorithms, three consensus candidate feature genes were identified, including PLIN2, LTA4H, and PPT1 (Fig 4F). A candidate feature model was then constructed based on these three genes. ROC curve analysis showed that the model had good discriminatory performance in both the training and validation cohorts, with AUC values of 0.838 and 0.833, respectively (Fig 4G). Further comparison of model-predicted KOA probabilities showed significantly higher values in the KOA group than in the control group (Wilcoxon test, p = 0.0045) (Fig 4H). Expression analysis showed that LTA4H and PPT1 were significantly upregulated in the KOA group in the training cohort and merged dataset, whereas PLIN2 was significantly downregulated in the KOA group. Similar trends were observed for all three genes in the validation cohort, although the differences did not reach statistical significance (Fig 4I). Further Reactome GSEA showed that LTA4H was mainly associated with pathways related to complement cascade activation, complement regulation, and FcεRI-mediated Ca² ⁺ mobilization. PPT1 was primarily enriched in pathways related to extracellular matrix organization, extracellular matrix degradation, MHC class II antigen presentation, and immune regulation. Pathways associated with PLIN2 downregulation were related to immune regulation, MHC class II antigen presentation, cell surface interactions at the vascular wall, and FCGR3A-mediated phagocytosis (Fig 4J).

**Fig 4:**
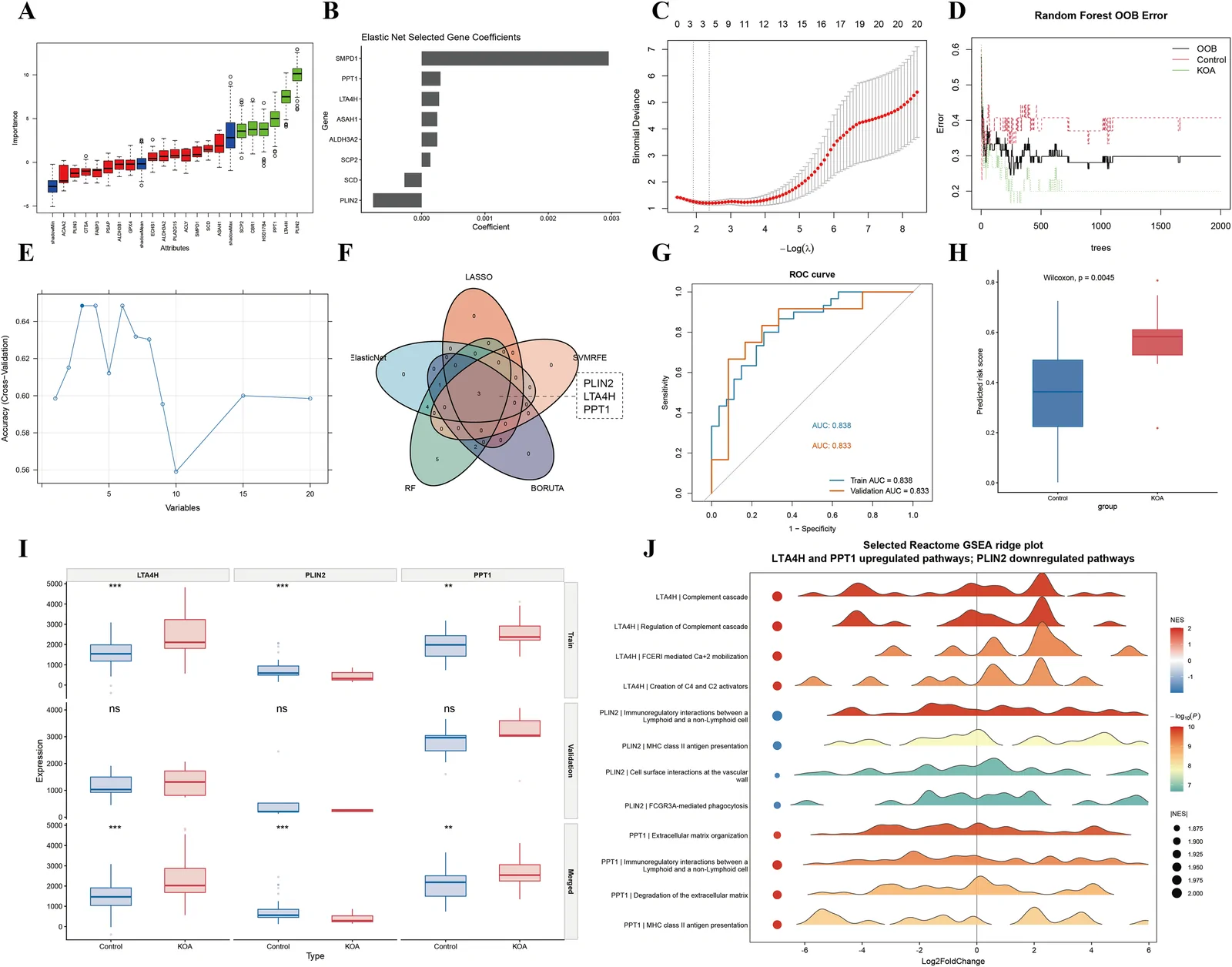
Machine-learning-based screening and functional analysis of KOA-related candidate feature genes. (A) Boruta algorithm evaluating the importance of the top 20 genes ranked by node degree in the PPI network; (B) Gene selection and regression coefficients in the elastic net model; (C) Cross-validation in LASSO regression for determining the optimal penalty parameter; (D) Out-of-bag error curve of the random forest model; (E) Cross-validation accuracy corresponding to different numbers of variables in SVM-RFE analysis; (F) Intersection analysis of genes selected by five machine-learning algorithms, including LASSO, elastic net, random forest, Boruta, and SVM-RFE; (G) ROC curves and corresponding AUCs of the candidate feature gene model in the training and validation cohorts; (H) Comparison of model-predicted KOA probabilities between the control and KOA groups; (I) Expression differences of the candidate feature genes LTA4H, PLIN2, and PPT1 in the training cohort, validation cohort, and merged dataset; (J) Reactome GSEA pathway enrichment analysis related to the candidate feature genes.

### Single-cell expression characterization of candidate feature genes and prioritization of the key candidate gene

To further characterize the single-cell expression profiles of the candidate feature genes identified by machine learning, we analyzed the distribution of LTA4H, PLIN2, and PPT1 expression in the scRNA-seq dataset. UMAP visualization showed that all three genes were expressed across distinct cell populations. LTA4H and PPT1 were mainly distributed in the myeloid-cell region, whereas PLIN2 showed relatively high expression in myeloid cells and mast cells (Fig 5A and 5B). Further comparison of gene expression in myeloid cells between the control and KOA groups showed that LTA4H and PPT1 were upregulated in the KOA group, while PLIN2 also exhibited a certain expression change trend (Fig 5C). After stratification according to the composite lipid metabolism score, all three genes showed varying degrees of increased expression in the score_UP group, suggesting their potential association with higher lipid metabolism-related transcriptional scores (Fig 5D). We then further assessed the correlations between the expression levels of these three genes and the composite lipid metabolism score in KOA myeloid cells. Spearman correlation analysis showed that LTA4H, PLIN2, and PPT1 were all significantly positively correlated with the composite lipid metabolism score. Among them, LTA4H showed the strongest correlation (Spearman R = 0.36, p < 2.2 × 10^ − 16), exceeding those of PLIN2 (Spearman R = 0.30, p < 2.2 × 10^ − 16) and PPT1 (Spearman R = 0.279, p < 2.2 × 10^ − 16) (Fig 5E and 5F). Therefore, LTA4H was computationally prioritized for subsequent cell-type-specific characterization.

**Fig 5:**
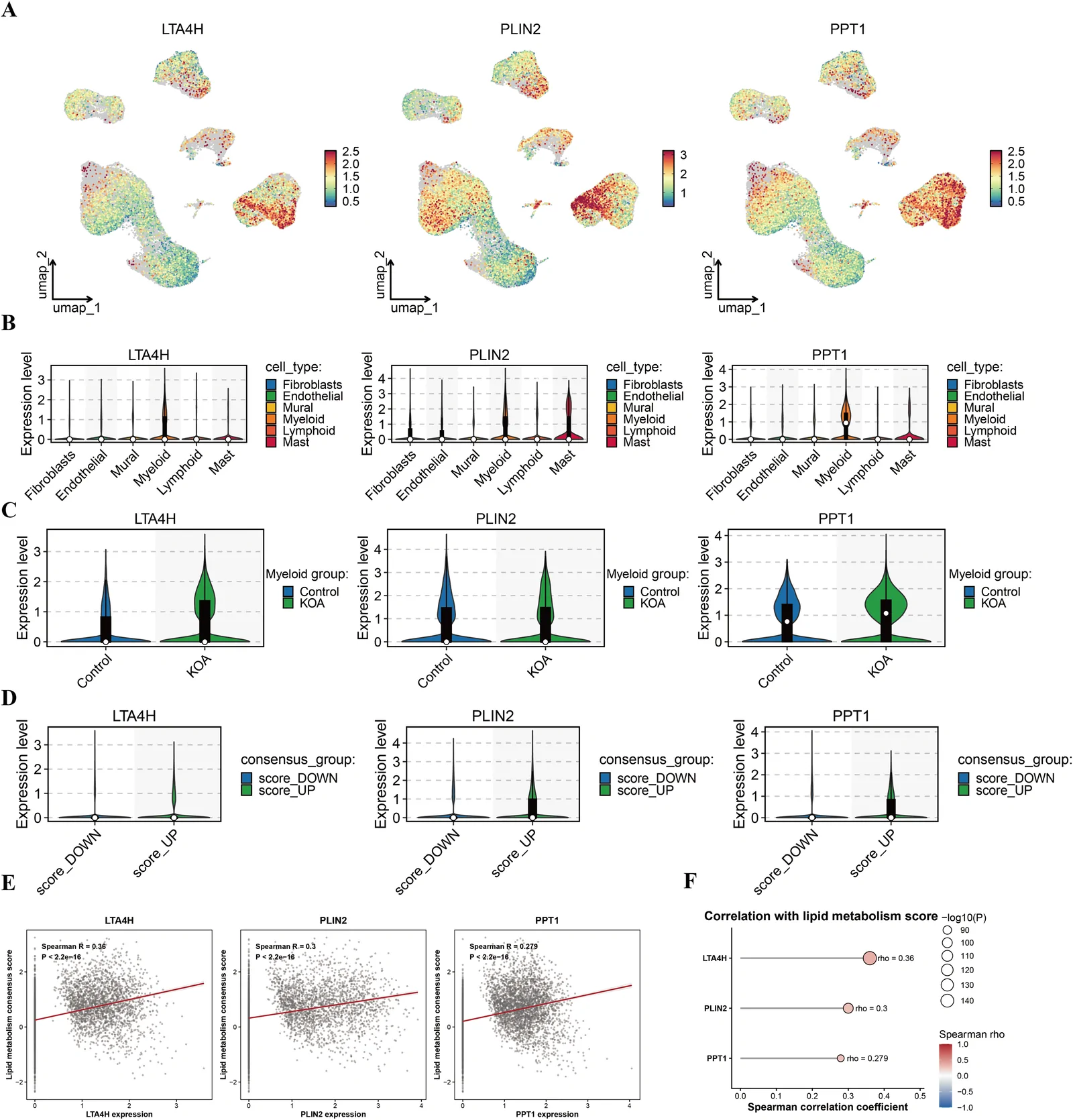
Single-cell expression characteristics of candidate feature genes and prioritization of the key candidate gene. (A) UMAP distribution of LTA4H, PLIN2, and PPT1 expression; (B) Expression levels of LTA4H, PLIN2, and PPT1 across different cell types; (C) Expression differences of the three genes between the control and KOA groups in myeloid cells; (D) Expression differences of the three genes between the high and low composite lipid metabolism score groups; (E) Spearman correlation analysis between the expression levels of the three genes and the composite lipid metabolism score; (F) Summary of Spearman’s ρ values for the three candidate genes in KOA myeloid cells (n = 4,823 cells).

### Cell–cell communication and pseudotime analyses of LTA4H-associated myeloid cells

To further characterize the functional features of LTA4H-associated myeloid cells, KOA myeloid cells were divided into LTA4H⁺Myeloid and LTA4H^−^Myeloid groups according to LTA4H expression status, followed by cell–cell communication analysis. The results showed that both LTA4H⁺Myeloid and LTA4H^−^Myeloid cells displayed extensive interactions with fibroblasts, endothelial cells, lymphocytes, mast cells, and mural cells (Fig 6A). Signaling pattern analysis further revealed differences among cell populations in both incoming and outgoing signaling patterns (Fig 6B and 6C). Ligand–receptor analysis indicated that LTA4H-associated myeloid cells were mainly involved in signaling axes such as CCL, CXCL, MIF, TNF, NAMPT, and LGALS9 (Fig 6D). We further performed pseudotime trajectory analysis of myeloid cells using Monocle 2. The results showed that myeloid cells were distributed across multiple States along the pseudotime trajectory and exhibited a continuous dynamic transition pattern (Fig 6E and 6F). Compared with the control group, the distribution of KOA cells along the pseudotime trajectory was altered (Fig 6G). Both LTA4H⁺Myeloid and LTA4H^−^Myeloid cells were distributed along the trajectory, and differences in LTA4H expression levels and the proportion of LTA4H-positive cells were observed across different States (Fig 6H–6J). As pseudotime progressed, the proportion of LTA4H-positive cells gradually increased and reached a relatively high level in late pseudotime States.

**Fig 6:**
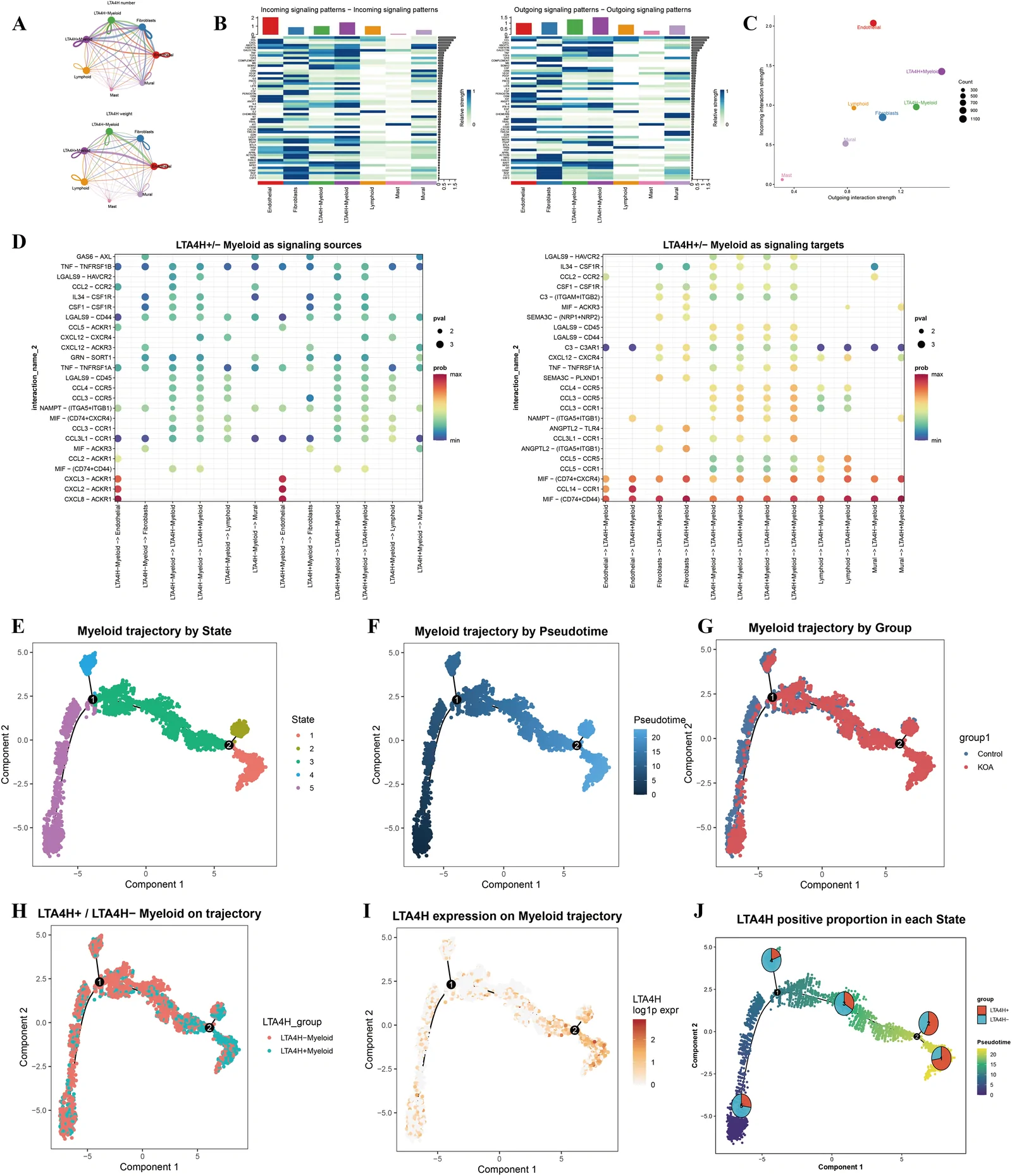
Cell–cell communication and pseudotime analyses of LTA4H-associated myeloid cells. (A) Overall cell–cell communication network between LTA4H⁺ Myeloid and LTA4H^−^ Myeloid cells and other cell types in KOA samples; (B) Heatmap of incoming and outgoing signaling patterns across cell populations; (C) Analysis of signaling sending and receiving strength across cell populations; (D) Ligand–receptor interaction analysis with LTA4H^+^/LTA4H^−^ Myeloid cells as signaling sources and target cells; (E) State distribution of myeloid cells along the pseudotime trajectory; (F) Pseudotime distribution of myeloid cells along the trajectory; (G) Distribution of myeloid cells from the control and KOA groups along the pseudotime trajectory; (H) Distribution of LTA4H⁺Myeloid and LTA4H^−^Myeloid cells along the pseudotime trajectory; (I) Distribution of LTA4H expression levels along the myeloid-cell pseudotime trajectory; (J) Proportion of LTA4H-positive cells across different States. CellChat analysis included 11,817 cells from three KOA samples, whereas pseudotime analysis included 9,512 myeloid cells from six biological samples.

### Myeloid-cell subclustering and inflammatory trajectory analysis

To further characterize myeloid-cell heterogeneity in KOA, myeloid cells were reclustered and annotated into subpopulations. UMAP visualization showed that myeloid cells could be further classified into five major subclusters: resident macrophages, monocytes, granulocytes, inflammatory macrophages, and dendritic cells (Fig 7A and 7B). Analysis of canonical marker gene expression demonstrated distinct expression patterns for each subcluster, supporting the reliability of the myeloid-cell subpopulation annotations (Fig 7C). Group-stratified UMAP visualization showed that both the control and KOA groups encompassed the major myeloid-cell subclusters, although differences in subcluster composition were evident (Fig 7D). Cell proportion analysis further revealed that the proportions of monocytes and inflammatory macrophages were relatively increased in the KOA group, whereas the proportion of resident macrophages was relatively decreased (Fig 7E). Further analysis of LTA4H expression across myeloid-cell subclusters showed that LTA4H was highly expressed in monocytes and inflammatory macrophages (Fig 7F). Monocytes and inflammatory macrophages were then subjected to Monocle 2 pseudotime analysis. The results showed that these two cell populations were continuously distributed along the pseudotime trajectory and exhibited a transition trend from monocytes toward inflammatory macrophages (Fig 7G and 7H). To determine whether this trajectory was accompanied by enhanced inflammatory features, we further examined the expression distributions of inflammation-related genes, including CCL3, TNF, IL1B, and OLR1, along the pseudotime trajectory. These genes displayed dynamic expression patterns and were relatively highly expressed in the late pseudotime region (Fig 7I). Consistently, the inflammatory score calculated based on these genes also indicated that cells in the late pseudotime stage exhibited higher inflammatory activity (Fig 7J). Further analysis of LTA4H dynamics along pseudotime showed that the proportion of LTA4H-positive cells gradually increased as pseudotime progressed and reached a relatively high level at the late pseudotime stage (Fig 7K).

**Fig 7:**
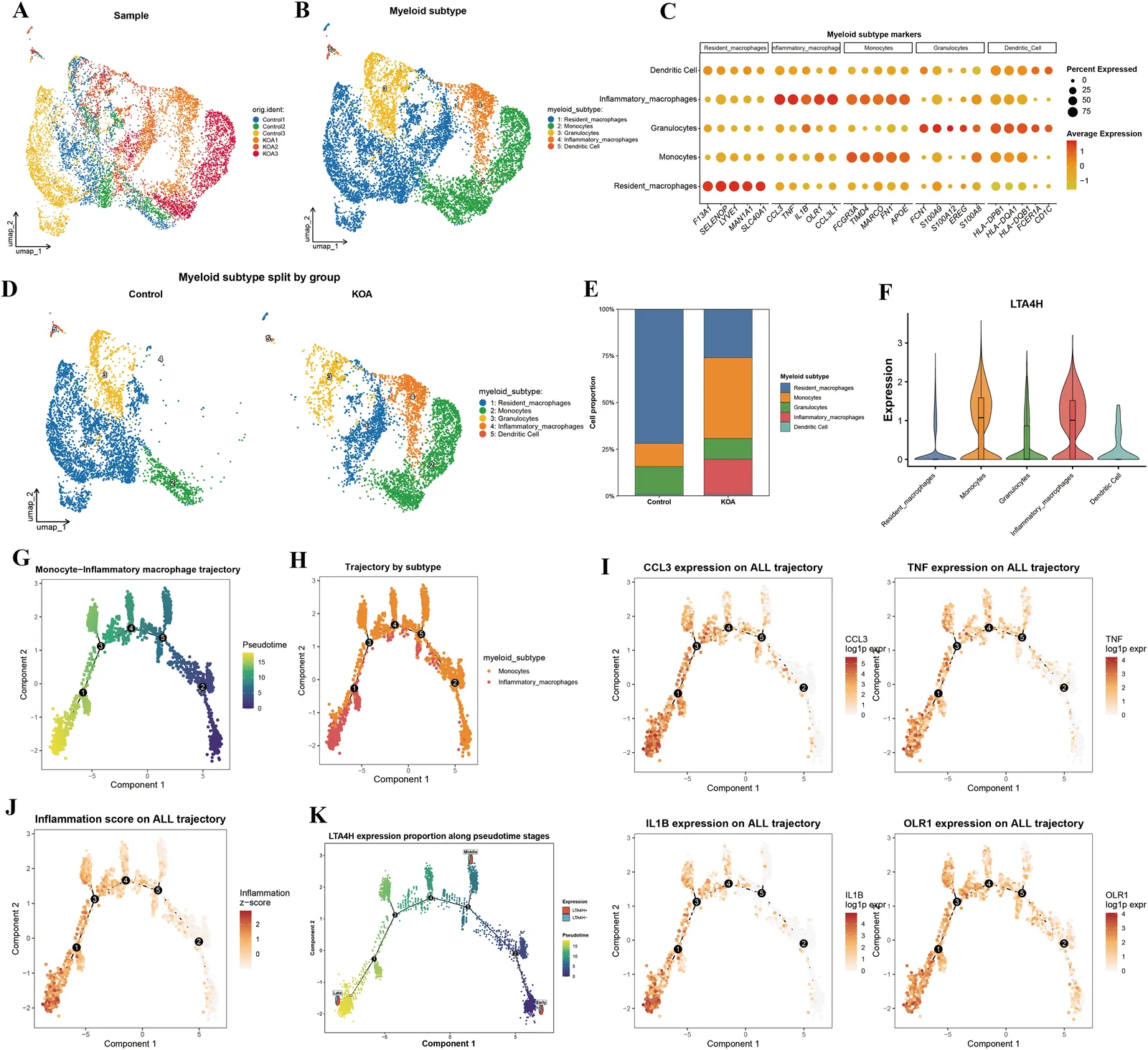
Myeloid-cell subclustering and inflammatory trajectory analysis. (A) UMAP distribution of myeloid cells by sample origin; (B) UMAP distribution of annotated myeloid-cell subclusters; (C) Dot plot showing the expression of canonical marker genes across myeloid-cell subclusters; (D) UMAP distribution of myeloid-cell subclusters in the control and KOA groups; (E) Compositional proportions of myeloid-cell subclusters in the control and KOA groups; (F) Expression levels of LTA4H across different myeloid-cell subclusters; (G) Pseudotime trajectory of monocytes and inflammatory macrophages; (H) Distribution of monocytes and inflammatory macrophages along the pseudotime trajectory; (I) Expression distributions of the inflammation-related genes CCL3, TNF, IL1B, and OLR1 along the pseudotime trajectory; (J) Distribution of the inflammatory score calculated based on inflammation-related genes along the pseudotime trajectory; (K) Changes in the proportion of LTA4H-positive cells along pseudotime progression. Subclustering included 9,512 myeloid cells, whereas pseudotime analysis included 3,579 monocytes and inflammatory macrophages from six biological samples.

### Effect of LTA4H in silico knockout on the regulatory network of inflammatory macrophages

To evaluate the regulatory role of LTA4H in inflammatory macrophages, we performed LTA4H in silico knockout analysis using scTenifoldKnk. The LTA4H-centered regulatory network showed that LTA4H was associated with multiple genes related to inflammation and macrophage function, including CX3CR1, S100A8, MARCO, CTSB, SELENOP, STAB1, and CCL2 (Fig 8A). Manifold alignment analysis showed that the regulatory network was markedly shifted after simulated LTA4H knockout compared with the baseline state (Fig 8B). Perturbation analysis revealed that multiple genes exhibited differential regulation in different directions after in silico knockout. Among them, LTBP2, CRIP2, SIX3, CX3CR1, DDX3Y, and CCL2 showed positive differential regulation, whereas THBS2, CCDC168, ATP5MGL, ZSCAN21, and NUP50-DT showed negative differential regulation (Fig 8C–8F). GO enrichment analysis showed that genes perturbed after LTA4H knockout were mainly enriched in functional terms related to leukocyte migration, leukocyte chemotaxis, phagocytosis, apoptotic cell clearance, granule lumen, vesicle lumen, chemokine receptor binding, and cytokine receptor binding (Fig 8G). These results suggest that, in inflammatory macrophages, perturbation of LTA4H may be associated with remodeling of regulatory networks involving multiple inflammation- and phagocytosis-related genes, with the affected genes primarily enriched in immune-inflammatory processes such as leukocyte migration and chemotaxis. These in silico findings suggest a potential regulatory role for LTA4H in inflammatory macrophages, while its causal regulatory effects require further mechanistic validation.

**Fig 8:**
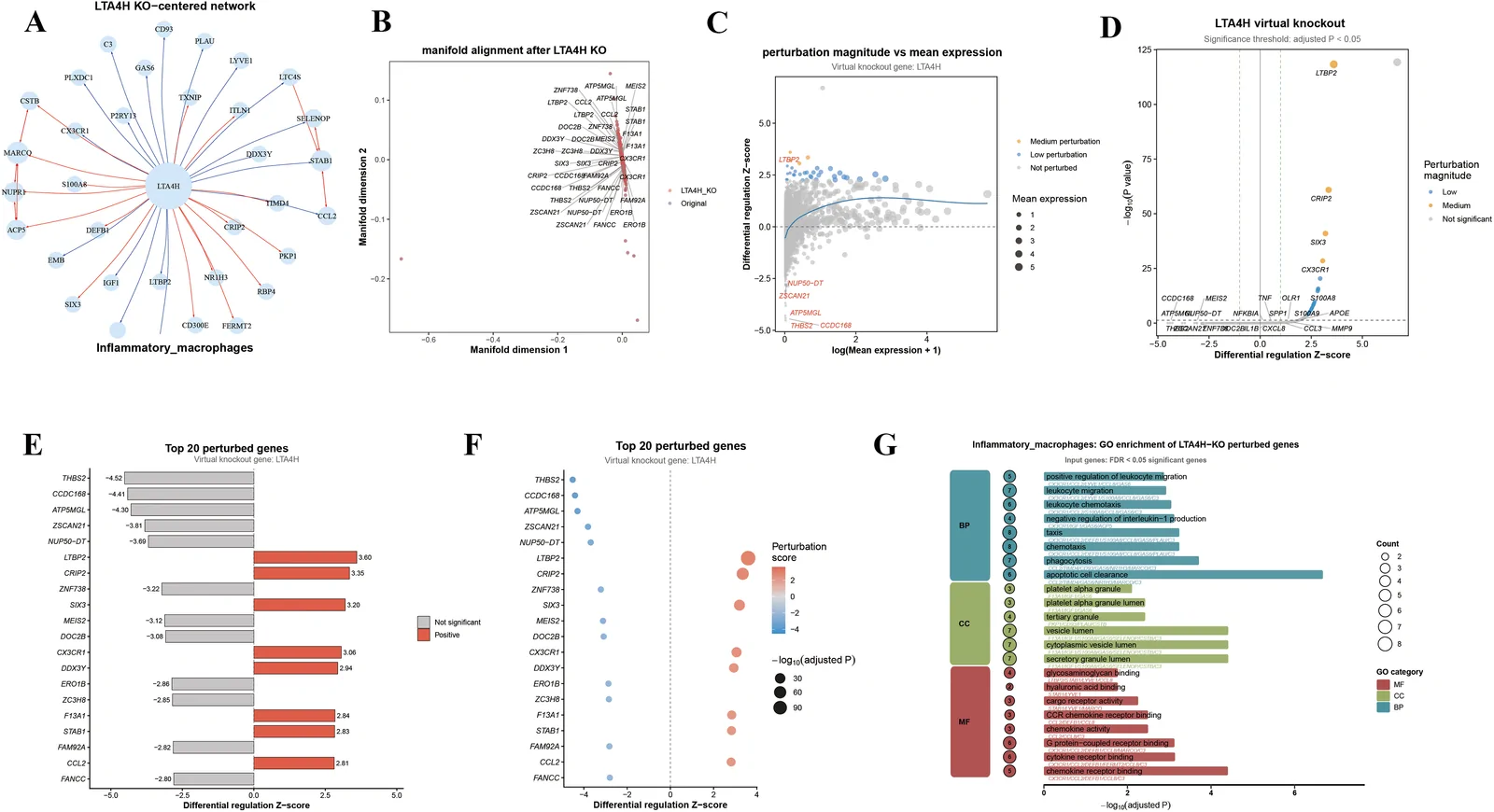
Effect of LTA4H in silico knockout on the regulatory network of inflammatory macrophages. (A) LTA4H-centered regulatory network in inflammatory macrophages under the in silico knockout framework; (B) Manifold alignment analysis of gene regulatory networks before and after LTA4H in silico knockout; (C) Relationship between mean gene expression and perturbation strength; (D) Volcano plot of gene perturbations after LTA4H in silico knockout; (E) Bar plot of the top 20 perturbed genes following LTA4H in silico knockout; (F) Perturbation strength and significance analysis of the top 20 perturbed genes; (G) GO functional enrichment analysis of genes perturbed after LTA4H in silico knockout. The analysis included 910 inflammatory macrophages from six biological samples.

### Experimental validation of LTA4H expression in rat knee synovial tissues

To further validate the computational findings, histological and immunostaining analyses were performed in a rat KOA model. Safranin O/Fast Green staining showed marked cartilage degeneration in the KOA group compared with the Sham group, characterized by disruption of the cartilage surface and reduced proteoglycan staining (Fig 9A). OARSI scores were significantly higher in the KOA group than in the Sham group (Fig 9B), indicating pronounced articular cartilage degeneration in KOA rats. Immunohistochemical staining was subsequently performed to assess LTA4H protein expression in knee synovial tissues. Compared with the Sham group, LTA4H-positive staining was markedly increased in the KOA group (Fig 9C), and quantitative H-score analysis further demonstrated significantly higher LTA4H expression in KOA synovial tissues (Fig 9D). These findings provide tissue-level experimental support for the increased expression of LTA4H in KOA synovium and are consistent with the preceding transcriptomic and single-cell analyses. Double immunofluorescence staining further showed spatial colocalization of LTA4H with CD68-positive macrophages in knee synovial tissues (Fig 9E), supporting the macrophage-associated localization of LTA4H.

**Fig 9:**
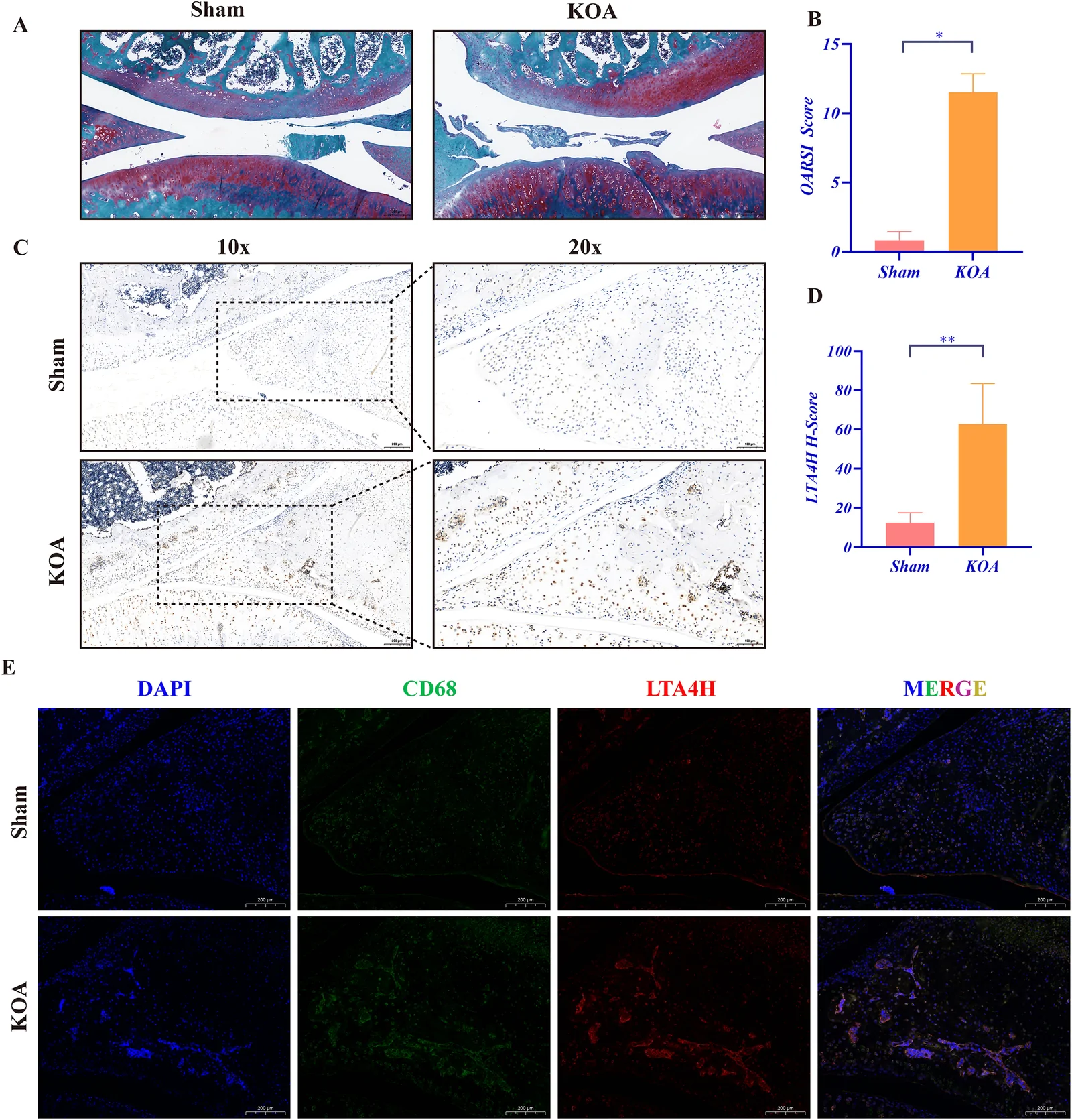
Experimental validation of LTA4H expression and macrophage-associated localization in rat knee tissues. (A) Representative Safranin O/Fast Green staining of knee joints from the Sham and KOA groups. (B) Quantification of cartilage degeneration using the OARSI histopathological score. (C) Representative immunohistochemical staining of LTA4H in knee synovial tissues from the Sham and KOA groups. (D) Quantitative analysis of LTA4H immunohistochemical staining using the H-score. (E) Representative double immunofluorescence staining showing the spatial colocalization of LTA4H with the macrophage marker CD68 in knee synovial tissues. Each point in the quantitative plots represents one animal (n = 4 per group). OARSI scores were compared using the Mann–Whitney U test, whereas H-scores were compared using an independent-samples t-test. *P < 0.05; P < 0.01.

## Discussion

Knee osteoarthritis (KOA) is a highly prevalent chronic degenerative joint disease characterized not only by cartilage degeneration and bone remodeling but also by synovial inflammation and immune-cell infiltration [12]. As a metabolically active intra-articular tissue, the synovium contributes to joint lubrication and cartilage nutrition and plays a critical role in inflammatory activation, immune signaling, and disease progression in KOA [25]. Recent studies have suggested that synovial tissues from patients with KOA exhibit not only prominent inflammatory-cell infiltration but also features of metabolic reprogramming, particularly alterations in lipid metabolic pathways [7]. Lipid metabolism is a fundamental metabolic process that provides cellular energy through lipid synthesis and degradation, regulates membrane composition, and generates bioactive lipid mediators involved in cellular homeostasis and immune regulation [26]. Dysregulated lipid metabolism may contribute to osteoarthritis pathology through multiple mechanisms. For example, lipid mediators derived from the arachidonic acid pathway, such as prostaglandins and leukotrienes, are increased in osteoarthritic joints and can activate inflammatory signaling pathways, including NF-κB. In addition, the accumulation of fatty acids and lipid oxidation products is closely associated with cartilage matrix degradation, oxidative stress, and synovial inflammation [27,28]. Previous lipidomic studies have revealed substantial remodeling of lipid profiles in synovial fluid and diseased tissues from patients with osteoarthritis. Compared with healthy controls, synovial fluid from patients with osteoarthritis contains increased levels of multiple sphingolipids and glycerophospholipids, and the abundance of these lipid species varies across disease stages. These alterations not only reflect lipid metabolic dysregulation but are also closely linked to joint inflammation and tissue degeneration [29,30]. However, because synovial tissue is composed of diverse immune and stromal cell populations, conventional bulk analyses cannot determine which cellular compartments predominantly contribute to lipid metabolic abnormalities. In the present study, we integrated five single-cell gene-set scoring algorithms to evaluate lipid metabolism-related transcriptional features in IPFP/synovium-derived cells and found that overall lipid metabolism-related scores were significantly increased in the KOA group, with high-scoring cells mainly distributed among myeloid cells and fibroblasts. Further stratified analysis showed increased lipid metabolism-related scores in myeloid cells, fibroblasts, and mast cells from the KOA group, whereas lymphocytes showed decreased scores. In parallel, myeloid-cell reclustering revealed increased proportions of monocytes and inflammatory macrophages in KOA. Previous experimental studies have shown that synovial macrophages are markedly expanded in osteoarthritic synovium and that these cells can polarize toward a pro-inflammatory M1-like phenotype under inflammatory microenvironmental conditions [31]. Together with our findings, these observations suggest that enhanced lipid metabolism-related gene programs in myeloid cells may be associated with altered inflammatory-cell composition and formation of a pro-inflammatory microenvironment within the KOA-associated IPFP/synovium compartment, whereas high scores in fibroblasts indicate that stromal cells within this compartment may also participate in local metabolic transcriptional remodeling.

In candidate-gene screening, we integrated lipid metabolism-related scoring, differential expression analysis, and PPI network information, ultimately identifying PLIN2, LTA4H, and PPT1 as consensus candidate feature genes selected across all five machine-learning algorithms. This intersection-based strategy reduced dependence on any single feature-selection method, although it may have excluded biologically relevant genes supported by only a subset of algorithms. Internal single-cell characterization further showed that these genes were generally upregulated in cells with high lipid metabolism scores. PLIN2 is a member of the perilipin family of lipid droplet-associated proteins and is involved in neutral lipid storage, lipolysis regulation, and lipid droplet homeostasis [30]. In the present study, PLIN2 was overall decreased in the bulk transcriptomic data and KOA myeloid cells, but was increased in cells with high lipid metabolism-related scores and positively correlated with the composite score. Previous studies using macrophage lipid-loading models have shown that PLIN2 deficiency reduces lipid droplet accumulation and cholesterol ester storage, but has limited effects on the elevation of inflammatory markers such as TNF-α and IL-6 [32]. Together, these observations suggest that PLIN2 may primarily reflect lipid-handling or lipid-droplet buffering states rather than a uniformly upregulated pro-inflammatory disease marker in KOA. PPT1 is a lysosomal palmitoyl-protein thioesterase responsible for removing long-chain fatty acids, such as palmitate, from proteins, thereby contributing to the degradation of lipid-modified proteins and maintenance of lysosomal lipid homeostasis [33]. In dendritic cells, PPT1 has been reported to regulate antigen degradation and endosomal acidification, thereby influencing antigen presentation and inflammatory cellular states, further supporting its role in immune-cell function and inflammatory signaling regulation [34]. Although direct experimental evidence linking PPT1 to KOA synovial inflammation remains limited, its involvement in lipid-related substrate processing and immune activation in immune cells is consistent with our observation that PPT1 was upregulated in myeloid cells with high lipid metabolism scores. These findings suggest that PPT1 may participate in lipid metabolic transcriptional reprogramming and inflammatory programs in KOA-associated IPFP/synovium-derived cells.

Among the three shared candidate feature genes, LTA4H showed the most prominent cell type specificity and disease relevance in this study. Internal single-cell characterization showed that LTA4H was predominantly enriched in myeloid cells, particularly monocytes and inflammatory macrophages, and was further upregulated in KOA myeloid cells. In addition, LTA4H expression was increased in cells with high lipid metabolism-related scores, and its correlation with lipid metabolism-related scores was stronger than those of PLIN2 and PPT1. These findings supported the computational prioritization of LTA4H for further cell-type-specific characterization of lipid metabolism-related transcriptional and inflammatory features in KOA myeloid cells. Mechanistically, LTA4H is a key enzyme in the arachidonic acid metabolic pathway and catalyzes the hydrolysis of the intermediate LTA4 to generate the pro-inflammatory lipid mediator LTB4, which promotes immune-cell chemotaxis, activation, and inflammatory signal amplification through BLT1/BLT2 [35,36]. Previous studies have shown that LTB4 can promote MAPK phosphorylation and enhance NF-κB-dependent inflammatory responses. In macrophages, the LTB4/BLT1 axis can further amplify NF-κB activation by relieving SOCS1-mediated inhibition of MyD88 [37,38]. These mechanisms are consistent with our finding that LTA4H-high myeloid cells were involved in inflammatory communication axes such as CCL, CXCL, MIF, and TNF, suggesting that LTA4H-associated myeloid cells may participate in enhanced inflammatory signaling within the KOA-associated IPFP/synovium compartment through coordinated lipid inflammatory mediator and chemokine networks. Further subclustering, pseudotime, and in silico perturbation analyses linked increasing LTA4H positivity to the monocyte-to-inflammatory-macrophage trajectory and to regulatory programs involving chemotaxis, phagocytosis, and inflammatory signaling. In inflammatory arthritis models, the LTB4/BLT1 axis has been shown to participate in IL-23-induced synovial inflammation and osteoclastogenesis through NF-κB [39]. Experimental osteoarthritis studies have also demonstrated that LTA4H is significantly upregulated in human osteoarthritic cartilage and in cartilage and synovial tissues from DMM mice, whereas pharmacological inhibition of LTA4H attenuates cartilage degeneration and synovial inflammation and upregulates cartilage-protective genes such as ACAN, COL2A1, and SOX9 [40]. These findings provide experimental support for the involvement of LTA4H in OA. Our single-cell analyses further localized LTA4H predominantly to myeloid cells, particularly monocytes and inflammatory macrophages, and linked its expression to lipid metabolism-related transcriptional features, inflammatory cell states, and myeloid-cell state transitions. Together, these observations refine the cellular context of LTA4H in KOA and support its prioritization for further mechanistic investigation. Consistent with these computational findings, tissue-level validation in the rat KOA model showed increased LTA4H protein expression in knee synovial tissues compared with the Sham group. Double immunofluorescence staining further demonstrated spatial colocalization of LTA4H with CD68-positive macrophages in knee synovial tissues, providing experimental support for the disease-associated upregulation and macrophage-related localization of LTA4H in KOA synovium.

This study has several limitations. First, the relatively small scRNA-seq cohort may affect the stability of cell-composition estimates and candidate-gene prioritization. Second, the analysis relied primarily on publicly available datasets and may therefore be influenced by differences in platform, batch, and sample source. In particular, the GSE216651 samples were annotated as joint-derived “IPFP/synovium cells,” precluding strict attribution of the observed single-cell states exclusively to synovial tissue. Furthermore, correlations of the composite lipid metabolism-related transcriptional score with nCount_RNA and nFeature_RNA indicate potential sensitivity to library complexity and transcript detection; therefore, the score should be interpreted as a transcriptional proxy rather than a direct measure of metabolic activity or flux. Finally, although the increased expression and macrophage-related localization of LTA4H were supported by tissue-level validation in rat knee synovial tissues, its causal role and underlying molecular mechanisms remain to be established. Future studies should validate these findings in larger human KOA cohorts and investigate the contribution of the LTA4H/LTB4 axis to inflammatory myeloid-cell states.

## Conclusions

This study integrated single-cell transcriptomic data from the IPFP/synovium compartment with bulk transcriptomic data from synovial tissues to characterize the cellular heterogeneity of lipid metabolism-related transcriptional features in KOA. Myeloid cells and fibroblasts emerged as major cell populations associated with these transcriptional alterations within the analyzed IPFP/synovium compartment. By combining machine-learning-based screening, single-cell expression characterization, cell–cell communication analysis, pseudotime inference, and in silico knockout analysis, LTA4H emerged as a computationally prioritized candidate with prominent myeloid-cell localization, particularly in monocytes and inflammatory macrophages, and was associated with lipid metabolism-related transcriptional features and inflammatory myeloid-cell states. These findings refine the cell-type-specific context of LTA4H in KOA, while tissue-level validation in rat knee synovial tissues further supports its increased expression and macrophage-related localization, providing a basis for further mechanistic investigation.

## Supporting information

S1 Fig Robustness and technical assessment of lipid metabolism-related transcriptional scoring. (A) Pairwise Spearman correlation matrix among AUCell, UCell, singscore, ssGSEA, and AddModuleScore scores calculated from the same lipid metabolism gene set. (B) Spearman correlations of the five individual scores and the composite score with nCount_RNA, nFeature_RNA, and mitochondrial transcript percentage. (C) Sample-level comparisons of the five individual scoring methods between the control and KOA groups; each point represents the median score for one biological sample. (D) Cell-type-specific KOA–control differences in sample-level median scores across the five individual scoring methods. Positive values indicate higher scores in KOA, whereas negative values indicate lower scores in KOA.
(TIF)

S1 Table Sample-level information, inclusion status, and analytical purposes of all GEO samples evaluated in this study. (XLSX)

S2 Table List of lipid metabolism-related genes included in this study. (XLSX)

S3 Table Canonical marker genes used for major cell-type annotation in the single-cell transcriptomic dataset. (XLSX)

S4 Table Machine-learning algorithm settings, feature-selection criteria, and final three-gene logistic regression model coefficients. (XLSX)

S5 Table Raw animal-level data for OARSI scoring and LTA4H immunohistochemical H-score analysis. (XLSX)
